# Discrimination of edge orientation by bumblebees

**DOI:** 10.1371/journal.pone.0263198

**Published:** 2022-06-16

**Authors:** Marie Guiraud, Mark Roper, Stephan Wolf, Joseph L. Woodgate, Lars Chittka

**Affiliations:** 1 School of Biological and Chemical Sciences, Queen Mary University of London, London, United Kingdom; 2 Drone Development Lab, Ben Thorns Ltd, Colchester, United Kingdom; Newcastle University, UNITED KINGDOM

## Abstract

Simple feature detectors in the visual system, such as edge-detectors, are likely to underlie even the most complex visual processing, so understanding the limits of these systems is crucial for a fuller understanding of visual processing. We investigated the ability of bumblebees (*Bombus terrestris*) to discriminate between differently angled edges. In a multiple-choice, “meadow-like” scenario, bumblebees successfully discriminated between angled bars with 7° differences, significantly exceeding the previously reported performance of eastern honeybees (*Apis cerana*, limit: 15°). Neither the rate at which bees learned, nor their final discrimination performance were affected by the angular orientation of the training bars, indicating a uniform performance across the visual field. Previous work has found that, in dual-choice tests, eastern honeybees cannot reliably discriminate between angles with less than 25° difference, suggesting that performance in discrimination tasks is affected by the training regime, and doesn’t simply reflect the perceptual limitations of the visual system. We used high resolution LCD monitors to investigate bumblebees’ angular resolution in a dual-choice experiment. Bumblebees could still discriminate 7° angle differences under such conditions (exceeding the previously reported limit for *Apis mellifera*, of 10°, as well as that of *A*. *cerana*). Bees eventually reached similar levels of accuracy in the dual-choice experiment as they did under multiple-choice conditions but required longer learning periods. Bumblebees show impressive abilities to discriminate between angled edges, performing better than two previously tested species of honeybee. This high performance may, in turn, support complex visual processing in the bumblebee brain.

## Introduction

Low-level feature detectors [1–5] such as edge orientation detector neurons [6] underlie visual object recognition, even in complex cognitive tasks [2, 6–10]. Roper et al. [11] demonstrated that it is possible to identify and discriminate a wide variety of complex visual patterns, using a low number of edge orientation detectors, without any need for storing “snapshot” visual memories. Differences in edge detection performance are thus likely to underpin interspecies differences in many visual discrimination tasks, so a detailed understanding of visual learning by bees and other insects will require an understanding of the limits of edge orientation detection. Bumblebees are a popular model for studies of insect visual learning [12, 13], providing significant advantages in that they can be bred and kept in indoor settings, which allows researchers to test them year-round in precisely defined laboratory conditions. There is currently no behavioural data available on how well bumblebees can discriminate between angled edges.

Wehner and Lindauer [14, 15] trained European honeybee workers (*Apis mellifera*) to collect food from feeders bearing either vertical, horizontal or 45° black bars. In tests, bees could discriminate the training pattern from one with only a 10° difference in orientation; at 8°, bees’ performance deteriorated; and they failed completely at 5°. Chandra et al. [6] trained eastern honeybee workers (*Apis cerana*) to feeders with black stripes of various angular orientations, presented on a white disk. Two tests were used: in one, the bees chose between two cues, one with stripes matching the trained orientation and one in a different orientation; the other test employed a multiple-choice paradigm, in which bees chose between the trained orientation and 11 deviations from it. Honeybees performed better in the multiple-choice situation, successfully choosing the trained orientation over alternatives rotated by 15°, whereas under the dual-choice conditions they only succeeded in differentiating between stripes that differed by 25°. This result gives a clear demonstration of the risks of inferring the limits of discrimination from behavioural performance, since such performance depends not just on what the bee sees, but also on the training procedure, as well as individual differences in motivation, cognition and, potentially, even “personality” [16]. There is mounting evidence that the conditioning procedure plays an important role in how animals perform in visual discrimination tasks [17–20]. Comparative methodological approaches allow us to understand the advantages and limits of each type of protocol [9, 21], and provide insight into the ways performance depends not just on sensory limitations but on how information is processed and used in different contexts.

We investigated the ability of bumblebee workers (*Bombus terrestris audax*) to discriminate between cues with differently oriented edges, in both a multiple-choice, meadow-like environment and in a dual-choice setup, to determine whether their performance differs from that of honeybees and how it is affected by the behavioural context.

## Methods

### Experiment 1: Multiple-choice (meadow paradigm)

#### Setup and pre-training

**Fig 1:**
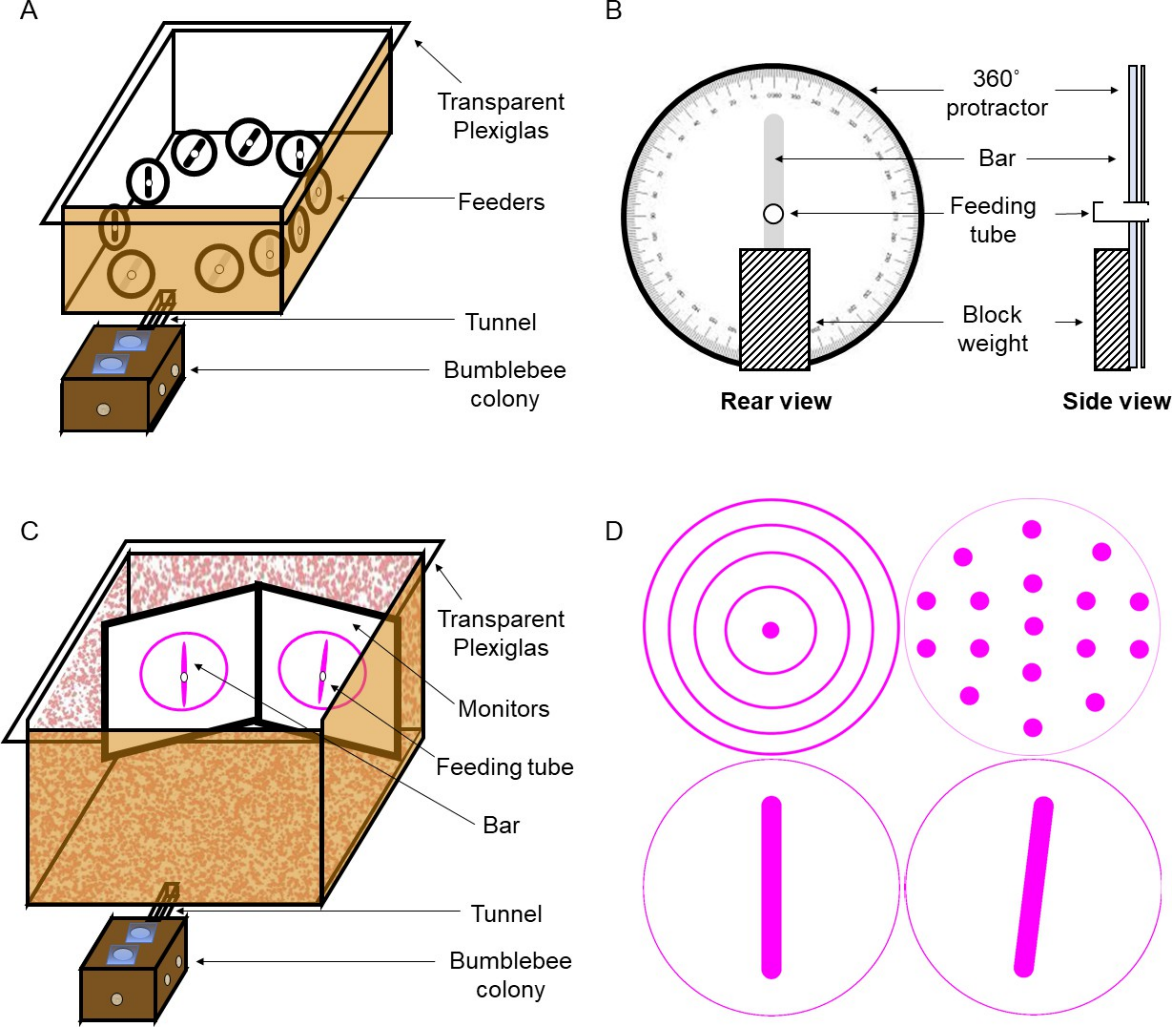
Experimental setup. **A:** Multiple-choice arena. A bumblebee colony was housed in a wooden box, connected to the experimental arena by a transparent tunnel. Ten feeders were presented in a circle configuration so that all were visible from the centre of the arena. **B:** Feeder. Each feeder consisted of a white paper disc with a printed black bar, and a feeding tube in the centre. A heavy block weight at the rear of the feeder supported a rotatable 360° protractor, allowing the bar to be rotated to the required angle. Five feeders, allocated at random, had the bar oriented to angle *A* and contained a drop of sucrose solution; the others were oriented to angle *B* and contained water only. **C:** Dual-choice arena. Two computer monitors were arranged at a 60° angle at the rear of the arena. Each presented a magenta bar in a white circle. A feeding tube was placed at the centre of each bar. One bar, allocated at random was oriented to angle *A* and the feeding tube contained a drop of sucrose solution; the other was oriented to angle *B* and contained water. **D:** Stimuli used in dual-choice experiments. Top row, two stimuli used during pre-training, one on each screen. Bottom row, example bar stimuli used for training and testing: left, 0° bar; right, 7° bar. All stimuli were displayed in magenta (RGB: 255, 0, 255) on a white background (RGB: 255, 255, 255).

We tested bumblebee workers from two commercially bred colonies (Biobest, Belgium). Each colony was kept in a custom-built wooden nest box (280 × 160 × 110 mm high) and connected via a Perspex tunnel to a foraging arena (700 × 700 × 400 mm high) with white painted walls and green laminated paper floor (Fig 1A). High frequency fluorescent lighting (TMS 24F lamps with HF-B 236 TLD ballasts, Phillips, Netherland; fitted with Activa daylight fluorescent tubes, Osram, Germany) were used to illuminate the foraging arena; the flicker frequency of the lights was ~42kHz which is well above the flicker fusion frequency for bees [22, 23].

During a pre-training phase, bees were allowed free access to the arena where they could forage on *ad libitum* sucrose solution (20% w/w) from ten feeders placed on the arena floor. Each feeder consisted of a sucrose-solution-filled feeding tube (Ø 7mm) placed horizontally at the centre of a vertically aligned, circular disk (Ø 110mm), made of laminated white paper. Each feeder was supported by an attached block weight (15 × 25 × 45 mm high) that allowed the feeder disk to be aligned perpendicular to the floor arena (Fig 1B). Bees foraging from the feeders were individually marked with number tags (Opalithplättchen, Warnholz & Bienenvoigt, Germany) for identification. Numbered bees that were observed to forage in the arena were allowed to advance to the individual training phase. Pollen was provided *ad libitum* into the colony.

#### Training and testing

During the training phase, bees were allowed to forage individually in the arena. Ten feeders (Fig 1B) were arranged in a circle, such that a bee could see all ten stimuli from the centre of the arena (Fig 1A). These feeders were identical to those used in pre-training, except that each white circle contained a single black bar (75 x 5 mm). Use of a 360° protractor attached to each feeding station, and spirit level on the arena base, allowed the experimenter to align the bar to precise angular orientations (Fig 1). Five feeders, randomly assigned, had the bar oriented at one angle, *A*, and provided 10μl sucrose solution (CS+: 30% w/w); the remaining five feeders had the bar oriented to a different angle, *B*, and were non-rewarding, filled with a 10μl water droplet (CS-). Feeding tubes were refilled from the rear, preventing sucrose or water from being deposited at the entrance of the tube.

In a pilot experiment, three bees very quickly learned to discriminate bars whose orientations differed by 15°, so we investigated whether they could discriminate a smaller difference. In experiment 1, we trained 25 bees to bar orientations differing by 7°. A range of bar orientations was used in order to examine discrimination performance across the full range of possible orientations. We started with four base angles at -60°, 0°, 45° and 90°. These were paired with an angle that differed by 7°, either clockwise or anticlockwise (e.g. 45° could be paired with 38° or 52°), giving 8 pairs of angles. We assigned pair of angles to each bee at random and further randomised which of the two angles in the pair was associated with the sucrose reward and which with water. There were thus 16 possible combinations of angles *A* and *B*, although only 12 were assigned to bees in practice. Table 1 gives details of how many bees were trained with each pair of angles.

**Table 1:** Number of bees tested with each pair of bar angles in experiments 1 and 2.

Base angle (°)	Direction of difference	Base angle assignment	Angle A (°)	Angle B (°)	No bees: Experiment 1	No bees: Experiment 2
**‒60**	+	A	‒60	‒53	1	0
**‒60**	+	B	‒53	‒60	2	0
**‒60**	‒	A	‒60	‒67	4	0
**‒60**	‒	B	‒67	‒60	2	0
**0**	+	A	0	7	1	1
**0**	+	B	7	0	0	0
**0**	‒	A	0	‒7	0	0
**0**	‒	B	‒7	0	0	0
**45**	+	A	45	52	4	0
**45**	+	B	52	45	3	0
**45**	‒	A	45	38	2	0
**45**	‒	B	38	45	0	1
**90**	+	A	90	97	1	1
**90**	+	B	97	90	1	1
**90**	‒	A	90	83	2	1
**90**	‒	B	83	90	2	1

Before training, all bees were removed from the arena and the setup was cleaned with 70% ethanol. The focal bee was allowed repeated access to the foraging arena until a total of 100 consecutive feeder choices were recorded. A choice was defined as a bee landing on a feeding tube, and for each choice we recorded whether the feeder was rewarding or unrewarding. After each foraging bout (consisting of multiple feeder choices, typically 4–10, continuing until the bee filled its crop, and returned to the nest box), all feeders were cleaned and the positions of rewarding and unrewarding were randomized before the bee was allowed to re-enter the arena. Rewarding feeders were refilled with 10μl sucrose solution each time a bee had drunk and departed from the feeder. Bees did not consume water from the unrewarding feeders, so they did not require refilling after each choice, but unrewarding feeders were cleaned and refilled after every foraging bout.

### Experiment 2: Dual-choice

#### Setup

We tested bumblebee workers from a third colony (Biobest, Belgium) on dual-choice tests, presenting the stimuli on two high-resolution, high-refresh-rate LCD computer monitors (Acer Predator GN246HLB, with 144Hz refresh rate, which is above the flicker fusion frequency of bees [23, 24]). These monitors were aligned and fixed in position, and software-generated oriented bars were used to ensure uniform angles.

A larger, wooden flight arena (1150 × 1300 × 500 mm high) was used to accommodate the monitors. The flight arena was covered with a red Gaussian random dot pattern (generated with custom MATLAB code [Mathworks Inc., Natick, USA]), printed onto white laser copy paper and laminated. At the top of the arena a fine fabric net was attached, this extended to the laboratory ceiling. High frequency fluorescent lighting (TMS 24F lamps with HF-B 236 TLD ballasts, Phillips, Netherland; fitted with Activa daylight fluorescent tubes, Osram, Germany) were used to illuminate the apparatus.

Two monitor screens were positioned at the rear wall of the flight arena and aligned at 60° angle from each other, allowing the bee to see both screens from the entrance of the arena (980 mm in front of the monitors). A transparent Plexiglas sheet was placed 15 mm from each monitor screen with a small hole (Ø 10mm) at the centre, leading to a feeding tube (Ø 8mm, 15mm long). As the tube was behind the Plexiglas from a bee’s point of view, it did not block any of her flight movements in front of presented stimuli and allowed us an unobstructed view of the bee’s movements.

#### Stimuli

Stimuli were created and displayed on the monitors using custom MATLAB code and the PsychToolbox [25]. Each monitor displayed a single open circle (Ø 260 mm) on a white background, with one or more shapes inside. All stimuli were magenta (RGB: 255, 0, 255), allowing observers to easily see the dark body of a bee in front of the monitor while still providing high levels of green-photoreceptor-contrast for the bee, which is required for edge detection and angle discrimination tasks [4, 26, 27]. During pre-training, one monitor showed a Ø 20 mm filled circle surrounded by four concentric circles (Ø 95, 160, 210 and 260 mm), while the other showed the Ø 260 mm circle containing an arrangement of 17 filled circles (Ø 20 mm; Fig 1D).

During training and tests, each monitor showed a circle containing a single bar (180 mm x 20 mm, with rounded ends; Fig 1D). Each rewarding bar orientation was paired with an identical, unrewarding bar, at a different angle. Rewarding stimuli (angle *A*) were assigned to 6 bees at random; the bar for the unrewarding feeder was rotated either +7° or -7°, relative to the rewarding stimulus (Table 1).

#### Pre-training

During the pre-training phase, bees were individually trained to feed from the feeding platforms and to learn that certain visual stimuli were associated with a sucrose reward. During this phase, no oriented bars were presented, but bees learned to discriminate between a pattern of concentric open circles, and an arrangement of 17 small, closed circles within the 260 mm outer circle (Fig 1D). One of these stimuli, assigned at random, provided 20μL of 50% (w/w) sucrose solution in the feeding tube (CS+); the other contained 20μL of water (CS-). The volume and sucrose concentration were increased from the 30% used in experiment 1 to maintain bees’ motivation to forage, since the rate at which they could visit feeders was reduced in the dual-choice setup.

A choice was recorded whenever a bee landed on one of the two feeding tubes. If the bee chose the tube in front of the unrewarding stimulus it was allowed to continue making choices until it landed on the rewarding feeder. Once the bee had located and consumed the 20μL sucrose solution from the rewarding feeder, it was captured in a transparent, ventilated transfer tube (Ø 30mm; 70mm long) and released again from the entrance of the arena to make another choice. Each foraging bout consisted of approximately four feeder visits, and lasted until the bee’s crop was full and it returned to the nest (mean crop capacity: 80 to 120μL [28]). Between foraging bouts, the feeders and surrounding Plexiglas were cleaned with 70% ethanol. The positions of the rewarding and unrewarding stimuli were pseudo-randomized between bouts (with no more than two consecutive bouts permitted with the same positions). Once a bee chose the rewarding pattern eight times out of the last ten feeder visits, it progressed to the experimental training phase.

#### Training and testing

The experimental training phase followed the same procedure as above. During each foraging bout, each of the two monitors showed an oriented bar (one feeder providing reward of 20μL of 50% sucrose solution w/w, and the other providing 20μL water). The location of the rewarding stimulus was pseudo-randomized for each foraging bout, and the feeders and surrounding Plexiglas were cleaned with 70% ethanol between bouts. Each bee was trained for approximately 130–190 choices, continuing until the bee achieved at least 80% correct choices over two consecutive 10-choice batches. If the bee did not achieve this 80% accuracy after 190 choices, the training was abandoned.

Once the bee achieved ≥80% correct choices in two consecutive batches of 10 choices, or failed to do so over 190 choices, it was subjected to a test bout. During tests, both feeding tubes contained a 20μL drop of water regardless of the stimulus. After each batch of 10 choices between unrewarding stimuli, the bee was given 10 choices under training conditions, with the trained stimulus again rewarded with sucrose, to maintain its motivation to visit the feeders. The number of choices was recorded for each bar during unrewarded test trials. The test bout ended when the bee no longer attempted to visit either feeding tube.

#### Analysis

We investigated whether bees in each experiment learned to discriminate between angled bars by testing whether the proportion of correct choices over the last 20 feeder visits by each bee, during training, was significantly greater than chance. We used a one-sample Wilcoxon signed-rank test for each experiment to compare the median proportion of choices for the CS+ to a chance level of 0.5 (using MATLAB’s *signrank* function).

The bees in experiment 2 were also tested on their performance in unrewarding probe trials. We tested whether their performance exceeded chance in this test, using another one-sample Wilcoxon signed-rank test, in which the proportion of choices for the CS+ was compared to a chance level of 0.5.

We investigated whether the orientation of the training bars in experiment 1 affected either the rate at which bees learned or their final performance. Bees were assigned to one of four orientation groups, in which we tested their ability to differentiate between bars at one of four angles (-60°, 0°, 45°, 90°) and bars ±7° from each of those angles (Table 1). Only one bee was tested in the 0°±7° group, so it was excluded from this analysis. The other three groups (-60°, 45°, 90°) contained 9, 9 and 6 bees, respectively.

To test whether experienced bees from each group differed in their ability to discriminate between angled bars, we used a Kruskal-Wallis test, in which the dependent variable was the proportion of choices for the CS+ over the last 20 feeder visits made by bees in each orientation group (using MATLAB’s *kruskalwallis* function).

To test whether there was a difference in the rate of learning between bees from each group, we used MATLAB’s *fitglme* function to create a generalized linear mixed effects model. The dependent variable was the arcsine-square-root transformed proportion of choices for the CS+ made by each bee in every block of 10 consecutive choices during training. It is normal for performance in learning tasks to plateau once the individual reaches a certain level of performance. To test only the bees’ performance during their learning phase, we calculated the proportion of correct choices over every block of 10 consecutive feeder visits and categorised individual bees as having learned the task if they made more than 80% correct choices over two consecutive 10-choice blocks. Only choices made up until an individual reached this threshold were analysed. The model included one continuous predictor, the number of 10-trial blocks experienced, and one categorical predictor, the angle group each bee belonged to. Bee identity was included as a random factor. The model tested for the main effect of block number and the interaction between block number and angle group. A significant effect of block number would demonstrate that discrimination performance improved as bees gained experience; a significant interaction term would demonstrate that the relationship between experience and performance was different for different groups and that bees trained on some angles learned at a faster rate than others.

Finally, we investigated whether there were differences between bees trained to discriminate 7° orientation differences under dual- and multiple-choice conditions in either final performance or rate of learning. We tested for an effect of experimental paradigm on the discrimination performance of experienced bees, by comparing the proportion of choices for the CS+ over the final 20 feeder visits made by bees in experiment 1 to those in experiment 2. Because there was a large difference in sample sizes between experiments (N = 25 and 6 for experiments 1 and 2, respectively), we used an exact permutation test (using the MATLAB function *permutationTest*, written by L. R. Kroll and available from https://uk.mathworks.com/matlabcentral/fileexchange/63276-permutation-test).

We tested whether there was a difference in the rate of learning between experiments 1 and 2, using another generalized linear mixed effects model. The dependent variable was again the arcsine-square-root transformed proportion of choices for the CS+ made by each bee in every block of 10 consecutive choices during training, up until they reached a threshold of 80% correct choices. The model included one continuous predictor, block number, and one categorical predictor, experiment number, and tested for the main effect of block number and the interaction between block number and experiment. A significant interaction term would demonstrate that the relationship between experience and performance was affected by differences between the experimental protocols.

All statistical tests were 2-tailed. All statistics were calculated in MATLAB (version R2021b; Mathworks Inc., Natick, USA).

## Results

### Experiment 1: Multiple-choice

**Fig 2:**
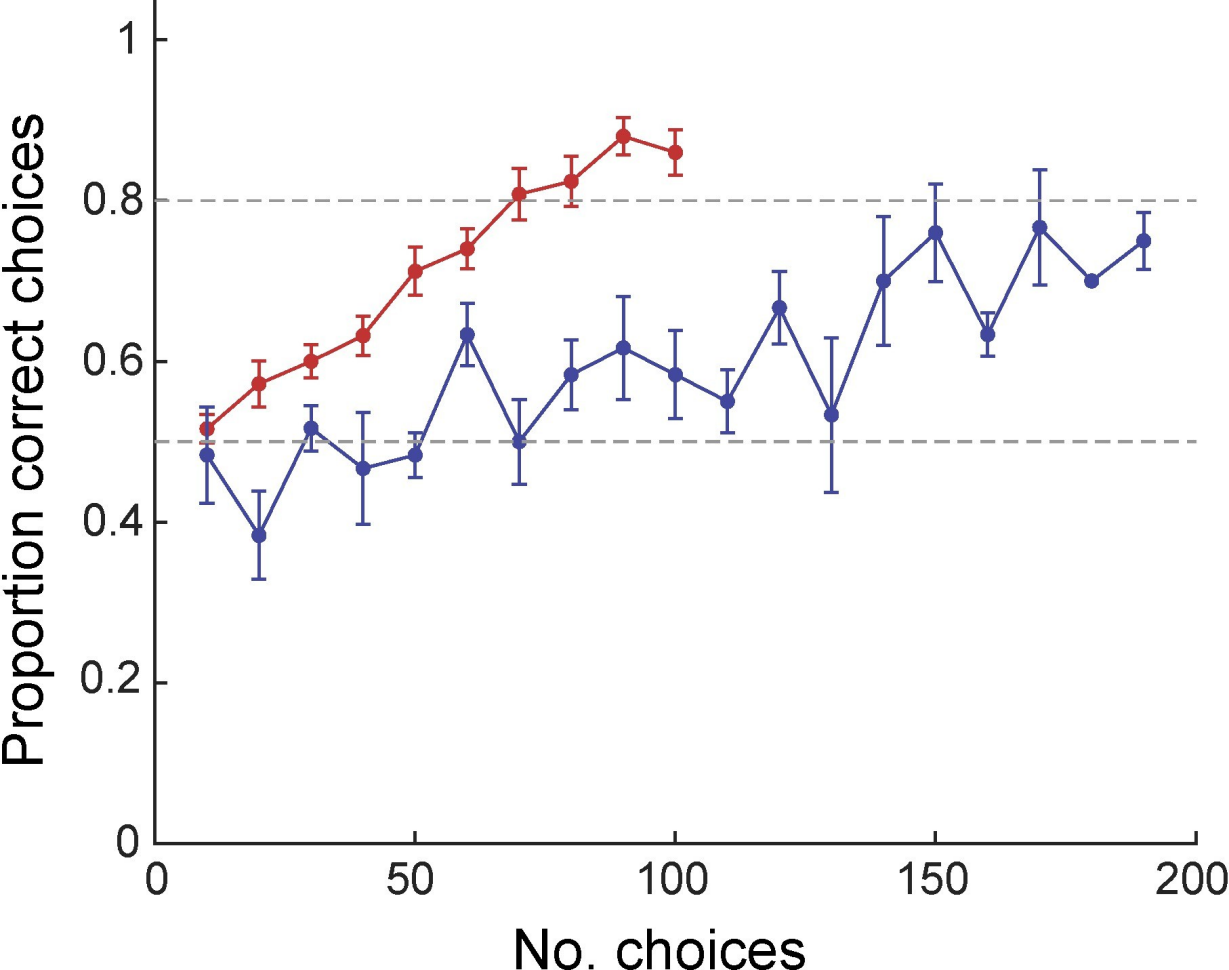
Learning curves for bees discriminating angles that differ by 7°. Each marker shows the mean proportion of choices (±S.E.) for the trained angle, *A*, across a block of 10 consecutive choices. Red circles, experiment 1 (multiple-choice, N = 25); blue circles, experiment 2 (dual-choice, N = 6). Dashed grey horizontal lines indicate a chance level of performance (0.5) and the threshold criterion we used as a proxy for the end of the learning period (0.8, but note that bees had to reach this proportion of correct choices over 20 feeder visits).

Bees trained to discriminate between angled bars whose orientation differed by 7° in a multiple-choice setting, learned to do so with high accuracy (Fig 2). The mean proportion of correct choices by all 25 bees in this group over their last 20 feeder visits was 0.870 ± 0.025 (means ± standard error, throughout), significantly greater than expected by chance (Wilcoxon signed-rank test: V = 325, N = 25, P <0.0001).

**Fig 3:**
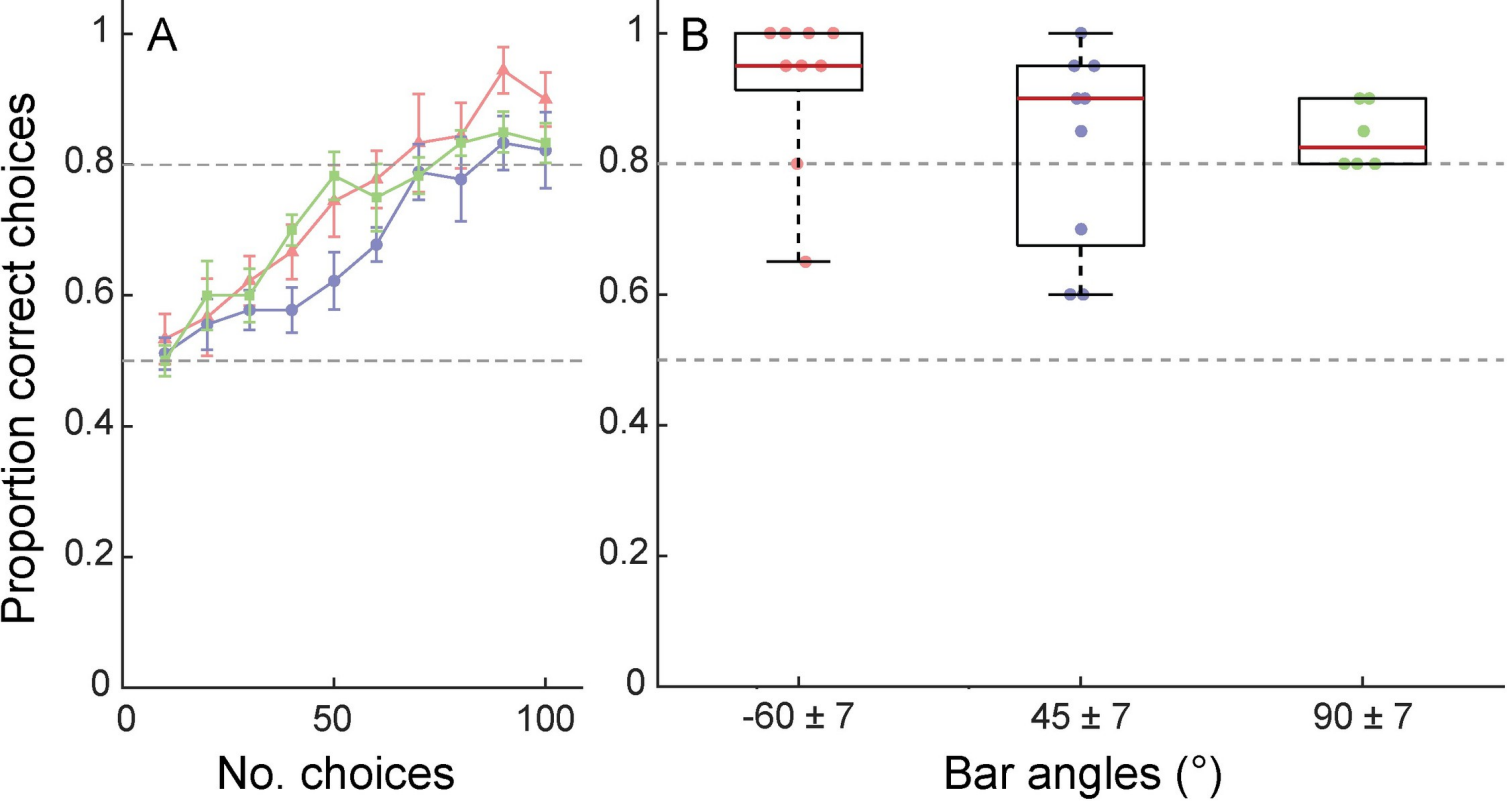
Angular discrimination is unaffected by training bar angles, in multiple-choice tests. **A:** Mean proportion of choices (±S.E.) for the trained angle, *A*, across each block of 10 consecutive choices for bees in experiment 1. Red triangles indicate bees that were trained to discriminate bars of -60° ± 7° (N = 9); blue circles, bees trained on 45° ± 7° bars (N = 9); green squares, bees trained on 90° ± 7° bars (N = 6). Dashed grey horizontal lines indicate a chance level of performance (0.5) and and the threshold criterion we used as a proxy for the end of the learning period (0.8 but note that bees had to reach this proportion of correct choices over 20 feeder visits). **B:** Proportion of choices for angle *A* (correct choices) across the last 20 feeder visits by bees trained to each group of angular orientations. Red lines indicate group median, boxes indicate the interquartile range and whiskers indicate the range. Filled circles show the proportion of choices for angle *A*, made by each individual. There are no significant differences between groups.

Although four groups of bees with different base bar orientations were used in the experiment, one group (0°±7°) contained only one bee, so we investigated whether bar orientation affected discrimination performance using the remaining three groups, only (-60°±7°, 45°±7°, 90°±7°; N = 9, 9 and 6 bees, respectively). There were no significant differences between the groups of bees trained on each of these three different base bar orientations in the number of correct choices over their final 20 choices (Kruskal-Wallis test: χ^2^_2,21_ = 4.98, P = 0.083; Fig 3).

We tested whether there was an effect of training bar orientation on the rate of learning using a generalized linear mixed effects model. There was a significant main effect of the number of 10-choice blocks of learning trials experienced on proportion of correct choices (F_1,156_ = 61.69, P < 0.0001; Fig 3A), demonstrating that discrimination performance improved throughout the learning period. There was no significant interaction between block number and training orientation group (F_2,156_ = 2.25, P = 0.108; Fig 3A), demonstrating that bar orientation did not affect the rate of learning.

### Experiment 2: Dual-choice

We also tested bees’ ability to discriminate a 7° angle variance using a dual-choice paradigm in which angled bars were displayed on two computer monitors. These bees also eventually reached a high level of performance: their mean proportion of correct choices over the last 20 feeder visits was 0.800 ± 0.034, significantly greater than expected by chance (Wilcoxon signed-rank test: V = 21, N = 6, P = 0.031; Fig 2).

The bees in this experiment were subjected to an unrewarded learning test after they had reached a threshold criterion of 80% correct choices over two consecutive 10-choice blocks, or after 190 choices in the case of two bees that never reached this threshold. Under these conditions, bees were as successful in discriminating angles as they were at the end of the training period, with a mean proportion of choices for the previously rewarding stimulus of 0.764 ± 0.065, significantly greater than expected by chance (Wilcoxon signed-rank test: V = 21, N = 6, P = 0.031).

### Differences between experiments 1 and 2

There was no significant difference in discrimination performance between bees in the two experiments over their final 20 choices (permutation test, P = 0.189).

A generalized linear mixed effects model demonstrated a significant effect of experience on discrimination performance across both experiments (F_1,255_ = 73.19, P < 0.0001; Fig 2). There was a significant interaction between block number and experiment (F_1,255_ = 26.49, P < 0.0001), demonstrating that bees in experiment 2 learned at a slower rate than those in experiment 1 (Fig 2).

We used a threshold of 80% correct choices over two consecutive 10-choice blocks to determine the end of the learning period for each bee. The large difference in the rate of learning between experiments is also reflected by the fact that 22 out of 25 bees in experiment 1 reached this threshold after a mean of 71.36 ± 3.37 choices (range: 40–100). By contrast, only four of six bees ever reached this level of performance in experiment 2, despite those that never reached the threshold experiencing nearly twice as many trials as those in experiment 1. The four that did reach 80% performance did so after 147.50 ± 8.54 choices (range: 130–170).

## Discussion

In this study, we demonstrate that bumblebees (*Bombus terrestris*) are able to discriminate between oriented bars with an angle difference of just 7°, in both a meadow-like multiple-choice, and a dual-choice scenario, and regardless of bar orientation. Similar levels of accuracy were eventually reached under both scenarios. This performance slightly exceeds that reported for European honeybees (*Apis mellifera*), which discriminated 10° differences with certainty and still showed some evidence of discrimination at 8°, in dual choice tests [14, 15]. By contrast, previous work has found that eastern honeybees (*Apis cerana*) could not be trained to discriminate angle differences below 15° in a multiple-choice setup, and below 30° under dual-choice conditions [6].

A number of studies have demonstrated an influence of the conditioning paradigm on bees’ behavioural performance in a variety of tasks [28–31]. We found that bees learned more slowly in a dual-choice than a multiple choice paradigm, demonstrating, that the training procedure can affect the outcome even of apparently simple perceptual tasks. In line with our results, Chandra et al. [6] found that *A*. *cerana* were able to discriminate smaller differences in orientation in a multiple-choice than a dual-choice paradigm, although it was unclear whether this reflected the maximum performance achievable under each scenario or whether the dual-choice bees were learning at a slower rate.

These results highlight the dangers of attempting to derive perceptual limits directly from behavioural studies. We can nonetheless draw some conclusions regarding the sensory/perceptual limitations faced by bumblebees. At the end of training, when bees had reached saturation-level performance, both groups of bees discriminated between edges differing by only 7° with a high level of accuracy (above 80% in both experiments), clearly demonstrating that they could perceive differences of at least that magnitude, and that this ability was not dependent on the specific conditions of one particular experimental paradigm.

Why might bees learn to discriminate angles more quickly in a multiple-choice experiment? One important factor may be the frequency with which bees were able to get feedback on their choices. In the meadow-like, multiple-choice experiment, bees visited several feeders during every foraging bout (round-trip from the nest to the flight arena and back), but in the dual-choice experiment they could make only one choice before being returned to the arena entrance. The comparative difference in how rapidly they can sample different options and rewards may account for the difference in learning speed. Alternatively, the lower rate of reward might have reduced bees’ motivation in the dual-choice experiment.

Wolf et al. [28] found that bumblebees could easily learn to discriminate between coloured feeders in a horizontal arrangement but ignored this information when the feeders were arranged vertically, and suggested that this was because the horizontal array was analogous to foraging in a meadow, where discriminating between different species of flower is important to maximise foraging success. The vertical array, by contrast may have been treated more like single inflorescences, or flowering trees, where discriminating between individual flowers is less important. It is possible that our multiple-choice experiment was a better analogue to a natural foraging scenario than the dual-choice experiment and thus promoted efficient discrimination learning.

There were a number of methodological differences between our experiments, so it is not possible to determine conclusively which affected the rate of learning. For example, the pre-training stage used different visual stimuli; the training stimuli were changed from black to magenta in the dual-choice experiment (to help observers track the bee’s position more effectively); and the volume and concentration of the sucrose reward was increased (to maintain bees’ motivation under conditions in which their rate of intake slowed); any of which may have affected motivation.

Chandra et al. [6] suggested that different motivations might explain the lower performance of *A*. *cerana* under dual-choice conditions: there was a 0.5 chance of reward from each feeder sampled, so it may have been efficient to sample all stimuli at random, rather than investing time and computation in discriminating between them. By contrast, in their multiple-choice experiment, the odds of success from random sampling were just 0.0833, so bees may have been motivated to invest in learning to provide a greater medium- or long-term rate of energy gain. The odds of success from random sampling were 0.5 in both of our experiments, however, so differences in expected reward from random sampling cannot account for the difference between paradigms.

The stimuli for our multiple-choice test were printed on paper, while the dual-choice test was presented on computer monitors, which have previously been found to make fine discriminations more difficult for bees [32]. However, Chandra et al. [6] reported differences between dual- and multiple-choice setups for eastern honeybees, even though their stimuli were printed on paper in both experiments, so the use of monitors is unlikely to account for the differences we observe.

We also cannot rule out a role for stochastic differences between the bees used in each experiment. Different colonies of bumblebees were used for the two experiments and intercolony variation in learning performance has been demonstrated in *B*. *terrestris* [33]. Future studies could fruitfully compare the performance of bees from the same colony under each paradigm; however, given that Chandra et al. [6] similarly found higher performance under multiple-choice conditions, we suggest that stochastic intercolony variation is unlikely to explain our results.

We did not measure any aspect of body size in our bees, so cannot investigate whether physiological differences may explain some of our observed variation in performance. Differences in body size are related to differences in eye size, number of ommatidia and facet size [34, 35], and recent work has suggested that there are intercolony differences in the scaling of body and eye sizes [36]. Differences in eye size predict visual acuity [34, 37]; could this be a limiting factor in angular discrimination? We did not impose any restrictions on our bees’ movements in the arena, particularly on their distance from the stimuli, so it is likely that smaller bees could compensate for any reduction in acuity by simply flying closer to the bars. We think it unlikely that differences in eye size could explain our results.

Body size in bumblebees has also been found to affect investment in learning [38], and how bees respond to the spatial layout of their environment [39]. Whether body size might affect the way in which bees approach an angular discrimination task, or their motivation or investment, deserves further exploration. Note, though, that in our study, bees in the dual-choice experiment eventually reached levels of performance indistinguishable from those in the multiple-choice experiment: if differences in physiology were responsible for differences in learning speed, bees were evidently able to compensate as they gained experience.

Even in a multiple-choice setup, the limit of angular discrimination previously reported for eastern honeybees [6] is double what we found for bumblebees, pointing to significant variation in visual discrimination ability between the two species. One explanation might be that visual-spatial resolution is constrained by eye optics: bumblebee workers are generally larger than honeybees and their larger eyes have both larger ommatidial facets and reduced interommatidial angles [34, 37, 40–42]. As a result, bumblebees have been found to have higher visual acuity than European honeybees (*Apis mellifera*), in several behavioural contexts [24, 34, 41]. Again, though, it seems likely that honeybees could compensate for lower acuity in these experiments by simply flying closer to the stimuli.

Supporting the argument that angle discrimination is not directly linked to visual acuity is the fact that European honeybees have been reported to show levels of angular discrimination only a little less than those we found in bumblebees [15]. The size and morphology of *A*. *mellifera* eyes lies between that of *A*. *cerana* and *B*. *terrestris* in many respects (such as ommatidial number and surface area [42, 43]), yet European honeybees performance was closer to that of bumblebees than this might predict. This suggests that optical resolution is not the limiting factor on honeybee performance. The specifics of flight behaviour are likely to play an important role, as are the characteristics of the neurons mediating edge orientation discrimination.

Our results and those of Chandra et al. [6], show that bees could discriminate angular differences regardless of bar orientation. Chandra et al. [6] used a mathematical model to suggest that a minimum of three orientation sensitive neurons would be required to account for this orientation indifference. However, this model assumed neurons with horizontal and vertical maximal sensitivities. Empirical work has subsequently identified two types of edge orientation sensitive neurons in the lobula, the third visual ganglion of the honeybee, with maximal sensitivity to edges angled at 115° and 220° from the vertical, respectively [44]. Roper et al. [11] presented computational-neuronal models based on the known properties of honeybee [44] and dragonfly neurons [45], which predicted performances remarkably similar to empirical honeybee results. Both models predicted that discrimination performance should be independent of the orientation of the training bars, as found in all three species of bees so far tested. These models support the hypothesis that angular discrimination performance is largely determined by the very limited number of orientation detector neurons identified in insects, and that these neurons’ angular tuning is adaptive in allowing uniform performance across a wide variety of orientations.
